# Discrete pre-processing step effects in registration-based pipelines, a preliminary volumetric study on T1-weighted images

**DOI:** 10.1371/journal.pone.0186071

**Published:** 2017-10-12

**Authors:** Nathan M. Muncy, Ariana M. Hedges-Muncy, C. Brock Kirwan

**Affiliations:** 1 Department of Psychology, Brigham Young University, Provo, Utah, United States of America; 2 Neuroscience Center, Brigham Young University, Provo, Utah, United States of America; UNITED STATES

## Abstract

Pre-processing MRI scans prior to performing volumetric analyses is common practice in MRI studies. As pre-processing steps adjust the voxel intensities, the space in which the scan exists, and the amount of data in the scan, it is possible that the steps have an effect on the volumetric output. To date, studies have compared between and not within pipelines, and so the impact of each step is unknown. This study aims to quantify the effects of pre-processing steps on volumetric measures in T1-weighted scans within a single pipeline. It was our hypothesis that pre-processing steps would significantly impact ROI volume estimations. One hundred fifteen participants from the OASIS dataset were used, where each participant contributed three scans. All scans were then pre-processed using a step-wise pipeline. Bilateral hippocampus, putamen, and middle temporal gyrus volume estimations were assessed following each successive step, and all data were processed by the same pipeline 5 times. Repeated-measures analyses tested for a main effects of pipeline step, scan-rescan (for MRI scanner consistency) and repeated pipeline runs (for algorithmic consistency). A main effect of pipeline step was detected, and interestingly an interaction between pipeline step and ROI exists. No effect for either scan-rescan or repeated pipeline run was detected. We then supply a correction for noise in the data resulting from pre-processing.

## 1 Introduction

Magnetic Resonance Imaging (MRI) has become a central tool in both research and medicine due to its ability to capture *in vivo* anatomic and functional data. Differences in structure and/or metabolism between groups can be strongly correlated with behavior performance [1–4], thus explaining the functions of the various regions of the cerebrum and cerebellum. Longitudinal studies give insight into neurodegenerative diseases and psychiatric disorders [5–12].

Central to these studies and diagnoses are various methods of interacting with the MRI data. Several studies [8, 9, 13, 14–19] have already investigated the effects of multiple software suites on a single dataset, finding that one often outperforms another in a certain regard. For instance, the FreeSurfer suite [20] is widely used for cortical and subcortical parcellation, yet recent studies have shown that Advanced Normalization Tools (ANTs) [21], a more recently developed suite of software, outperforms FreeSurfer in certain aspects [17, 18, 22].

Pre-processing steps like rigid-body transformations, field-inhomogeneity corrections, and skull-stripping are quite common [7, 18, 23–26]. While their effects on the data, such as adjusting the position of the data within the field-of-view or the distribution of the histogram, are well known and needed for the sake of both algorithmic robustness and multi-modal approaches, the impact of such effects are not well investigated; few studies have investigated different pre-processing steps and quantified their impact on the data [27, 28], and have done so while also investigating varying software and pipelines. Further, few studies have quantified the variance of multiple intra-subject scans resulting merely from pre-processing rather than some other independent variable [29–32]. Consequently, no consensus on how each pre-processing step impacts the downstream output has been established.

As it is unknown how each step within a single pipeline influences the overall output of the pipeline, different researchers with similar questions could potentially achieve significantly different results that are not a product of the independent variable under investigation, but rather the result of different pre-processing steps (let alone software). Differing pipeline steps may introduce an unknown amount of noise into the data even if their pre-processing algorithms are based in the same suite of software. This may be especially true if the various pre-processing steps add different amounts of noise or variance as a difference of only 1 to 2% between groups may be considered significant [33].

It is therefore the aim of this paper to assess the impact of common pre-processing steps on scan-rescan variance and anatomic volume. We analyzed the estimated volumes of bilateral putamen, hippocampus, and middle temporal gyrus in 115 participants, and varied pre-processing steps to include images left in original space, rotated and cropped images, N4-bias correction, and skull-stripping of the images. We hypothesize that each different pre-processing step will have a differential impact on the region of interest (ROI) estimated volume.

## 2 Methods

### 2.1 MRI acquisition

Hosted cross-sectional data from the Open Access Series of Imaging Studies (OASIS) project [34] were used in this study. 115 participants whose age ranged between 20 and 29 (female = 68, mean age = 22.8 ±2.48) were selected from the entire database, as we intended to only include healthy young adults. All participants were scanned with a T1-weighted MPRAGE sequence using following parameters: slice thickness = 1 mm, TR = 1900 ms, TE = 2.26 ms, field of view = 218 × 250, voxel size = 1 × 0.977 × 0.977 mm, acquisition matrix = 256 × 215, flip angle = 9°. As each participant contributed three or four scans, the first three scans of each participant were used in this study.

As all images were hosted in a 16-bit big-endian Analyze 7.5 format, images were converted into NIfTI and MGZ formats via ***mri_convert*** for processing in ANTs- or FreeSurfer-based pipelines, respectively, detailed below.

### 2.2 FreeSurfer

FreeSurfer 6.0 (https://surfer.nmr.mgh.harvard.edu) [20, 35] was used to render hippocampal, putamen, middle temporal gyrus, and total brain volumes in all subjects. All scans were segmented using the FS software with the command ***recon-all –subjid subjDir –all –sd /path/to/workDir -notal-check –cw256***. Volumes were then extracted from the aseg.stats, lh.aparc.DKTatlas.stats, and rh.aparc.DKTatlas.stats output files. FreeSurfer output was also hosted by OASIS, and the same data were extracted from the hosted FS data; we expect our data to differ from the OASIS FS data, as we are using an updated version of the software. While it is not the aim of this paper to compare between diverse pipelines, but rather individual steps within a single pipeline, FS was included due to its ubiquity in order to have a reference point for our findings. Further, referencing the DKT atlas with produce complimentary labels to the protocol below.

### 2.3 Template construction

Twenty representative participants were selected in a pseudo-random fashion from the 115 participants (female = 10, mean age = 22.8 ±2.5) in order to construct a study-specific template. First, scans were warped into a standardized coordinate space (MNI) [36, 37, 38] using a nonlinear diffeomorphic normalization algorithm supplied by ANTs [14] with the following command:

***ANTS 3 –o <prefix> -i 100x100x100x20 –t SyN[0*.*1]–r Gauss[3*,*0*.*5]–m CC[<template*.*nii*.*gz>*, *<input*.*nii*.*gz*, *4*, *4]; WarpImageMultiTransform 3 <input*.*nii*.*gz> <output*.*nii*.*gz> Warp*.*nii*.*gz Affine*.*txt –R <template*.*nii*.*gz>***.

Second, warped scans were then skull-stripped, as we intended to render both a head (brain with meninges, skull, etc) and a brain (all non-brain information removed) template, for use with images containing head, or just brain, information, respectively. Skull-stripping was done utilizing ANTs and referencing the OASIS-30 template hosted at http://www.mindboggle.info/data.html via ***antsBrainExtraction*.*sh –d 3 –a lab_template*.*nii*.*gz –e ref_template*.*nii*.*gz –m probability_mask*.*nii*.*gz –f registration_mask*.*nii*.*gz –o <prefix>***. Third, structural scans in MNI space were then used to construct a lab-specific head and brain templates, following previous research [17, 21], via ***buildtemplateparallel*.*sh –d 3 –m 30x90x30 –t GR –s CC –c 2 –j 8 –o <prefix> -z mni_icbm152_template*.*nii <structural_mni_scans*.*nii*.*gz>***. This resulted in templates derived from the statistical average of all input scans. Fourth, the lab-specific head template was segmented according to the Desikan-Killiany-Tourville protocol using the Joint Label Fusion toolkit and referencing the OASIS-TRT-20 dataset [22, 39–41]:

***antsJointLabelFusion*.*sh –d 3 –t <template*.*nii*.*gz>–o <prefix> -p prior%04d*.*nii*.*gz –c 5 –j 4 –g atlas_1/<atlas*.*nii*.*gz>–l labels_1/<labels*.*nii*.*gz> … -g atlas_20/<atlas*.*nii*.*gz>–l labels_20/<labels*.*nii*.*gz>***

This produced ROI-specific probabilistic priors in template space, for use in anatomic segmentation. The ROI priors were produced only in head template space, as both the head and brain templates existed in the same space, and in order to reduce template-mask confounds during segmentation. Finally, priors for skull-stripping participant scans were constructed in template head space. This was first accomplished by skull-stripping the head template by referencing the OASIS-30 priors via

***antsCorticalThickness*.*sh –d 3 –a <moving*.*nii> -e <fixed*.*nii> -t <brain_mask*.*nii> -m <probability_mask*.*nii> -f <extraction_mask*.*nii> -p priors%d*.*nii*.*gz –o <prefix>***.

Next a probability mask was constructed with ***SmoothImage 3 <binary_mask*.*nii> 1 <probability_mask*.*nii>***, an extraction mask was constructed with ***c3d <binary_mask*.*nii> -dilate 28x28x28vox –o <extraction_mask*.*nii>*** [42], and a registration mask constructed via ***c3d <binary_mask*.*nii> -dilate 1 18x18x18vox –o <registration_mask*.*nii>***.

### 2.4 Pre-processing participant scans

**Fig 1:**
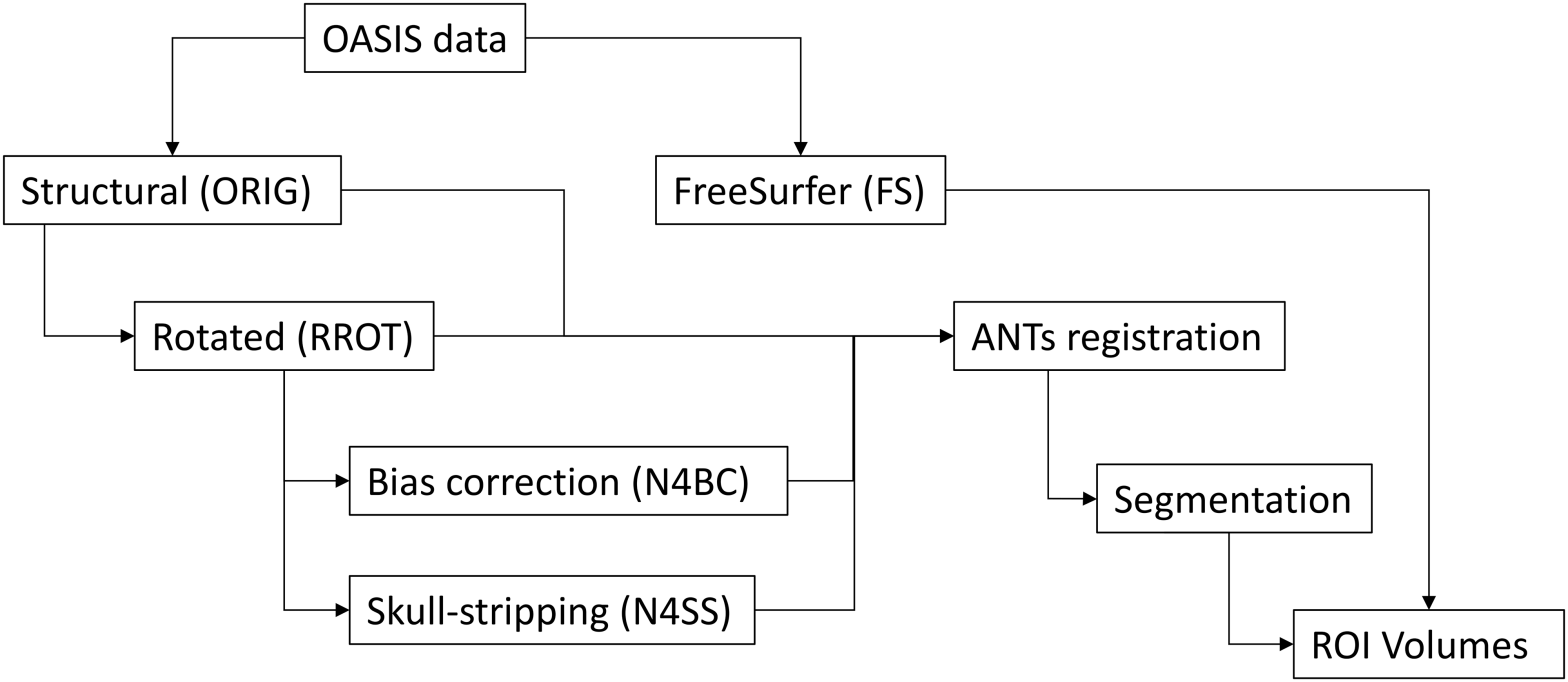
Illustration of the pre-processing pipeline. OASIS data are first converted into structural NIfTI (ORIG) and MGZ files. MGZ files are processed via FreeSurfer (FS). NIfTI files are rotated (RROT) which are then N4-bias corrected (N4BC). Also, RROT files are skull-stripped (N4SS; which has its own bias correction step). The output of each pipeline step is registered with a segmented template, and ROI masks are warped from template to participant pipeline-step space. ROI volumes are then extracted from the segmentation masks. FreeSurfer pre-processing is entirely self-contained, and produced its own set of ROI volumes.

As we intended to investigate whether or not pre-processing MRI data has an impact on volumetric output, scans from each of the participants were then pre-processed in a stepwise fashion such that the output of a previous step became the input for the subsequent step. Common pre-processing steps [23–25, 29, 43, 44] were selected from the literature; all pre-processing and segmentation was conducted on all three scans contributed by each participant. First, images converted from the Analyze format to NIfTI format where left in native space (ORIG). Second, a rigid-body transformation utilizing six degrees of freedom rotated (RROT) the structural scans into approximate template space via ***antsRegistrationSyNQuick –d 3 –f <template*.*nii> -m <input*.*nii> -t r –o <prefix>***. RROT differs from ORIG in the location of information within the domain, which may impact matrix-based calculations performed in a diffeomorphic registration. Third, RROT scans were then used to produce N4-bias corrected scans (N4BC) [45] via the ANTs-based toolkit ***N4BiasFieldCorrection –d 3 –i <input*.*nii*.*gz> -s 4 –c [50x50x50x50*,*0*.*0000001]–b [200]–o <output*.*nii*.*gz>***, where care was taken to use the same parameters and arguments found in the ***antsBrainExtraction*.*sh*** script. This step corrects non-uniform image intensities of the same tissue class which result from field inhomogeneities. For example, gray matter in one region may have a very similar voxel intensity to white matter of another region due local signal variation resulting from field inhomogeneities. Correcting for such variance produces tissue classes that have similar intensity signatures. Finally, scans were skull-stripped (N4SS), a process which involves the removal of non-brain tissues (e.g., the durae), skull, and scalp, ***via antsBrainExtraction*.*sh –d 3 –a <input*.*nii*.*gz>–e <template*.*nii*.*gz>–m <probability_mask*.*nii*.*gz>–o <prefix>***. Additionally, as the skull-stripping script performs the N4-bias correction prior to segmentation, RROT scans were used as the input files in this step in order to avoid repeated bias corrections. Thus, each participant would be measured by the ORIG, RROT, N4BC, and N4SS scans produced by the previous steps, in addition to the FreeSurfer (FS) measures (Fig 1).

### 2.5 Segmentation

Segmentation of the participant scans (ORIG, RROT, N4BC, and N4SS) was then conducted in two steps. First, ANTs was used to register each scan to the template, resulting in a calculation of the bilateral diffeomorphic deformation of both template and participant scan to a common midpoint. This was done with the command ***ANTS 3 -o Reg -i 100x100x100x20 -t SyN[0*.*1] -r Gauss[3*,*0*.*5] -m CC[template*.*nii*.*gz*, *input*.*nii*.*gz*,*4*,*4]***. Care was taken to register non-skull-stripped scans (head) with the head template, and skull-stripped (brain) scans with the brain template. Upon completion, the calculations were then used to warp 6 probabilistic segmentation labels from template to participant space using a nearest-neighbor interpolation. The labels included two subcortical structures (bilateral hippocampus and putamen) and one cortical structure (bilateral middle temporal gyrus). The hippocampus was selected due to its difficulty in segmentation, whereas the putamen was considered easier to segment being clearly separated from nearby structures by white matter. The middle temporal gyrus was selected in order to include a cortical measure. Segmentation of these various regions of interest (ROIs) occurred via ***WarpImageMultiTransform 3 <ROI_label*.*nii*.*gz> <output*.*nii*.*gz> -i Affine*.*txt InverseWarp*.*nii*.*gz –R <input*.*nii*.*gz>—use-NN***. Upon review, and in accordance with the literature [46], these ROI masks in participant space were then thresholded and binarized via ***c3d <input*.*nii*.*gz>–thresh 0*.*5 1 1 0 –o <output*.*nii*.*gz>***. In total, six measures (bilateral hippocampus, putamen, middle temporal gyrus) were produced for each of the pre-processing steps (ORIG, RROT, N4BC, N4SS, and FS) for each participant (n = 115), yielding a large number of observations. Further, repeated scans for each participant contributed additional measurements, which will be used to assess scan-rescan variance. ROI volumes were extracted via ***c3d <input*.*nii*.*gz> -dup –lstat***, while Dice similarity coefficients [47] were extracted via ***c3d <input1*.*nii*.*gz> <input2*.*nii*.*gz> -overlap <label>***.

### 2.6 Total brain volumes

In order to perform a ratio correction, total brain volumes were calculated for all scans for each participant, using both FreeSurfer-and ANTs-based tools. Calculations of total brain volumes of the mgz files were pulled from the aseg.stats file, and total brain volumes of the NIfTI files were derived from the binary BrainExtractionMask produced by ***antsBrainExtraction*.*sh*** thereby giving six total brain volumes (TBV) for each participant, for each scan. Each ROI was then converted into a ratio of TBV in order to account for hidden confounds such as scanner warmth.

### 2.7 Repeated pipeline runs

In order to assess the consistency of the various software used in sections 2.4–6, the data from all participants were processed via the pipeline described above a total of five times. This will help determine if the various software interacts consistently or idiosyncratically with the MRI data, as it is assumed that the algorithms perform consistently. Thus it can be determined whether the significant variance, if any, is the product of the pre-processing software, scanner inconsistencies, or the pre-processing steps within the pipeline. All scripts used for in this study are available at https://github.com/nmuncy/Preproc_Effects.

## 3 Results

### 3.1 Within-subject repeated measures

All analyses were performed on the volumetric output of six brain regions (left and right putamen, hippocampus, and middle temporal gyrus). To control for potential confounds (e.g. the size and sex of the participant, daily hydration levels, scanner warmth, etc.), each volumetric output was corrected by the total brain volume prior to analyses. The total brain volumes were calculated separately for the FS- and ANTs-based pipelines, and these values were used as denominators for their respective volume measurements, producing a ratio.

To determine the effect of pre-processing steps, we performed a multivariate repeated measure analysis on the six corrected ROI volumes and the five different pre-processing steps for each of the 115 participants at an α = 0.05 level. That is, the regions of interest and the different methods are the two within-subject factors. The Hotelling’s *T*^*2*^*(4*,*113)* value (a generalized *F* statistic of a repeated measure that determines the discrepancy of sample volume mean and hypothetical mean) of 7349.77 exceeded the critical value (CV) of 10.08 (generalized partial *η*^2^ = 0.25), indicating that regional ratios of at least one of the pre-processing pipelines differed significantly from the others across the tested regions. In other words, there are significant differences in regional ratios that are dependent on how the scans are pre-processed. Additionally, a main effect of ROI was detected (*T*^*2*^*(5*,*113)* = 10648.60, CV = 11.91, generalized partial *η*^2^ = 0.33). Most interestingly, there is an interaction effect of pre-processing step and ROI (*T*^*2*^*(20*,*113)* = 3637.80, CV = 40.47, generalized partial *η*^2^ = 0.13). This means that pre-processing steps differentially affect the volumetric measurement of ROI for at least one of the steps. Finally, there was no sex effect (*F* = 0.10, *p* = .75).

As the main effects and interaction of multivariate analysis were significant, we then performed *t*-tests for each ROI to find which pre-processing steps significantly differ from each other. Though it is unnecessary to perform any multiple comparison corrections because the multivariate analysis was significant [48, 49], we performed the very conservative Bonferroni correction (Table 1). In this follow-up analysis, we see the differential effects of pre-processing on the regions. For the left and right putamen, three step comparisons produced significantly different volumes after a Bonferroni correction (Table 1). ORIG differed significantly from both RROT and N4BC in both hemispheres, and the right hemisphere differed between the N4SS and FS. For the left and right hippocampus, ORIG produced significantly different volumes from RROT and N4BC, but not from N4SS, and N4SS only differed from FS. The RROT and N4BC steps also produced significantly different volumes in the hippocampi. For the left and right MTG, all step comparisons significantly differed except RROT vs N4BC. Please note that these results only apply when each ROI is corrected by total brain volume produced by each step. Without this correction, there are more ROI by step interactions (see supplementary).

**Table 1:** Ratio pairwise comparisons of pipelines.

	LPut		RPut		LHip		RHip		LMTG		RMTG	
Comparison	t	*p*	t	*p*	t	*p*	t	*p*	t	*p*	t	*p*
ORIG vs RROT	10.99	**<.0001**	11.20	**<.0001**	12.61	**<.0001**	12.33	**<.0001**	11.16	**<.0001**	13.48	**<.0001**
ORIG vs N4BC	13.2	**<.0001**	13.40	**<.0001**	15.50	**<.0001**	14.57	**<.0001**	8.65	**<.0001**	13.50	**<.0001**
ORIG vs N4SS	-0.81	.42	-0.84	.40	-0.79	.43	-0.87	.39	-3.56	**.0005**	-2.73	**.007**
RROT vs N4BC	2.30	.02	2.57	.01	3.94	**.0001**	4.85	**<.0001**	-1.08	.28	1.70	.09
RROT vs N4SS	-1.50	.14	-1.52	.13	-1.52	.13	-1.56	.12	-4.28	**<.0001**	-3.64	**.0004**
N4BC vs N4SS	-1.62	.11	-1.65	.1	-1.69	.09	-1.77	.08	-4.26	**<.0001**	-3.74	**.0003**
N4SS vs FS	-1.67	.10	-5.78	**<.0001**	-34.68	**<.0001**	-30.52	**<.0001**	-18.83	**<.0001**	-14.76	**<.0001**

Significant *t*-values show which method of pre-processing produces regional ratios that differ significantly from one another, i.e. which two pre-processing steps produce dissimilar ratios for each region of interest. FS is only compared to N4SS as they have a similar pipeline. Also, these findings indicate that as data move along the overall pipeline that is commonly performed (Orig → RROT → N4BC → N4SS), volumes significant differ depending on the brain region of interest. Bolded values are significant at the Bonferroni corrected value of 0.05/7. L = left, R = right, Put = putamen, Hip = hippocampus, MTG = middle temporal gyrus.

### 3.2 Repeated pipeline runs

The first scan from each participant was processed using the various pre-processing pipelines four more times. These repeated pipeline runs allowed us to investigate whether the significant mean ROI by step differences detected in Section 3.1 are the result of the different pre-processing steps, or whether they were the result of the pre-processing algorithms creating variance that did not previously exist in the actual data. In other words, repeated iterations of the pipelines can tell us either (a) that the difference is the result pre-processing steps interacting consistently with varying noise inherent in the data, or the result of each step producing a different but consistent amount of noise, or (b) that the steps produce differing and idiosyncratic amounts of noise in the data. We assumed that if the repeated iterations of the pipelines produced statistically identical volumes, then the volumetric differences calculated were the result of one or both parts of (a). Unfortunately, this study cannot tease apart the two parts of (a).

To analyze the five pipeline runs, we performed another multivariate repeated measure analysis after repeatedly processing all data with FS and each of the ANTs-based pipelines. Both methods produced statistically similar volumes for all the five runs (*T*^*2*^*(4*,*114)* = 4.08, CV = 10.08, generalized partial *η*^2^ = 0.0002). This implies that the algorithms are consistently interacting with the data.

As an additional summary measure for the multiple runs, we provided Dice similarity coefficients (DSC) [50, 51] between the runs for each participant and each brain region as shown in Table 2. DSC is a measure of overlap in space, where DSC is a distance measure of the overlap of two ROI labels divided by the total. Values for the DSCs range from 0 to 1, where scores ≥ 0.7 are considered “good” and a score of 1 indicates perfect agreement [52, 53]. All steps had very high similarities.

**Table 2:** Dice similarity coefficients.

Step	LPut	RPut	LHip	RHip	LMTG	RMTG
ORIG	0.999 (*0*.*0001*)	0.999 (*0*.*0002*)	0.999 (*0*.*0002*)	0.999 (*0*.*0002*)	0.999 (*0*.*0008*)	0.999 (*0*.*0007*)
RROT	0.999 (*0*.*0003*)	0.999 (*0*.*0006*)	0.999 (*0*.*0007*)	0.999 (*0*.*0008*)	0.999 (*0*.*0033*)	0.999 (*0*.*0048*)
N4BC	0.999 (*0*.*0006*)	0.999 (*0*.*0008*)	0.999 (*0*.*0011*)	0.999 (*0*.*0011*)	0.999 (*0*.*0068*)	0.999 (*0*.*0062*)
N4SS	0.998 (*0*.*0022*)	0.998 (*0*.*0022*)	0.997 (*0*.*0036*)	0.997 (*0*.*0034*)	0.985 (*0*.*0210*)	0.987 (*0*.*0170*)
FS	1.000 (*0*.*0000*)	1.000 (*0*.*0000*)	1.000 (*0*.*0000*)	1.000 (*0*.*0000*)	1.000 (*0*.*0000*)	1.000 (*0*.*0000*)

Mean Dice coefficients across runs and participants, mean (SD). All pre-processing steps have extremely high similarities.

### 3.3 Scan-rescan analysis

To test for scanner consistency, we processed the three repeated scans taken from the OASIS dataset on the 115 participants through the same FS- and ANTs-based pipelines. Once again, we performed a multivariate repeated measure analysis, now with three within-subject factors (day, method, and ROI). The *T*^*2*^*(2*,*114)* value of 0.46 (critical value of 6.21 and generalized partial *η*^2^ = 0.00004) indicates the repeated scans of the same participant produced consistent volumes for all the regions tested.

Thus, the significant ROI volumetric differences detected in Section 3.1 is in fact an effect of pre-processing the data, and not due to the algorithms inconsistencies (Section 3.2) nor is the differences the result of an artifact resulting from a scan-by-pipeline-step interaction (Section 3.3).

### 3.4 Percent variability error correction

As the different steps in pre-processing give different mean corrected volumes, this variability must be taken into account before comparing between groups’ regions of interest. One method to account for these differences is to find the percent variability error in scan-rescan for each pipeline step in order to find the error bounds. To calculate the percent variability error, we followed the method described by Tustison et al., [22] and averaged the absolute difference for scan-rescan for each participant in each method. That is, if *T*_*ijk*_ is the *k*th scan in step *j* for participant *i* (*i = 1*,*…*,*N*), then the percent variability error is

$$\varepsilon_{ij}=\frac{1}{N}\sum_{i=1}^{N}\sum_{k=2}^{n}\frac{2\left|T_{ij1}-T_{ijk}\right|}{\left(T_{ij1}+T_{ijk}\right)*\left(n-1\right)}$$ (1)

The percent variability error for each step is given in Table 3. Corrections for all six brain regions are included. Each value indicates the percentage of the specific region value to add to or subtract from each method’s corrected volume when comparing group averages, longitudinal measurements, or the different pre-processing methods. In other words, if researchers are using the N4BC ratio pipeline, their left hippocampal corrected volumes should be considered as X ± 0.0151X (PVE = 1.51) or as

$$\frac{regional\;volume}{total\;brain\;volume}\pm \frac{regional\;volume}{total\;brain\;volume}*0.0151$$ (2)

That is, if the hippocampal volume was 0.22% of the total brain volume for the N4BC, then this value should be treated as 0.22% ± 0.003%, thereby giving the range of left hippocampal volume percentage as 0.217%– 0.223%.

**Table 3:** The mean percent variability error for each method.

Step	LPut	RPut	LHip	RHip	LMTG	RMTG
ORIG	1.00	1.14	1.36	1.17	1.40	1.18
RROT	1.40	1.31	1.51	1.41	1.56	1.43
N4BC	1.48	1.43	1.51	1.46	1.60	1.45
N4SS	3.41	3.26	3.37	3.55	3.64	3.71
FS	2.42	2.41	2.35	1.86	3.09	2.58

Because the pre-processing methods are significantly different from each other, each step in each region needs a correction to be able to compare different methods. Though the percent variability error may appear small, the values in this table are for the regional volume divided by the total brain volume.

While these percentages may not seem significant, consider the percentages in terms of actual volume. In this study, the average total brain volume produced by N4BC was 1,513,592 mm^3^. The left hippocampal volume range of 0.217%-0.223% becomes 3284.5–3375.3 mm^3^, translating to a range of about 90.8 mm^3^. This is relevant in the comparison of groups using the same pipeline, or of various studies using differing pipelines, where the upper bounds of one group or pre-processing pipeline may actually fall within the lower bounds of another. Similar to a 95% confidence interval when estimating parameters, this range given by the percent variability error takes the variance of the pre-processing method into account. This is so a comparison between groups may be representative of independent variables and not simply the differences of pre-processing methods or other confounds.

## 4 Discussion

### 4.1 Summary

This study investigated the effects of certain pre-processing steps in registration-based volumetric ROI segmentation. Analyses showed a main effect of pre-processing step, indicating that the various steps investigated significantly altered the ROI-corrected volume. Further, and importantly, an interaction of pre-processing step and ROI was discovered, indicating that the alterations in ROI-corrected volumes were not consistent across different brain regions. The significantly different corrected volumes found between the pre-processing steps may be the result of the algorithm of each pre-processing step interacting with the data in a consistent but unique fashion, although it is possible that the algorithms were in fact interacting with each scan idiosyncratically. We tested this assumption by running all data through the same pipeline four additional times; no effect of run was detected. Additionally, it was possible that the volumetric differences resulted from inconsistent scanner output, thereby producing an interactive effect between the pre-processing step and MRI noise. To test this, three scans of each participant were processed through the same pipeline steps; an effect of repeated scans was not detected, indicating consistent MRI output. As such, we concluded that it was the pre-processing steps that significantly changed the ROI-corrected volumes, step to step, and not the algorithms performing inconsistently or variance from the scanner itself. Finally, we supplied a correction for each step and for each ROI to account for the noise in the data resulting from pre-processing.

### 4.2 Limitations

First, only a few specific pre-processing steps were investigated in this study, and as such the generalizability of our findings are quite restricted. In this preliminary study, an effort was made to investigate whether or not unaccounted-for and significant variance existed in registration-based volumetric data, and whether the noise was a result of pipeline algorithms, scanner output, or pre-processing protocol. As such, we used pre-processing steps based within the same software suites in order to reduce potential confounds. While significantly different volumes, resulting from pipeline steps, were detected, such findings are constrained to the parameters of our study. It is reasonable, however, that significant variance resulting from pre-processing will be detected in other pipelines, in accordance with our findings, as pre-processing algorithms alter the data directly. Further, and more importantly, our attempt to quantify variance that is unaccounted for was done in order to address a deficit in the literature: while numerous studies have investigated differences between differing pipelines [17, 18, 22, 27, 45, 54–61], these do not investigate the effects of pre-processing on the data within a single pipeline. If, as was detected in this study, pre-processing steps significantly change the data, then it may not be meaningful to compare studies which differ in as little as a single pre-processing step, and may be even less meaningful to compare studies using different pre-processing software. Second, while we investigated discrete steps within a single pipeline, we did not investigate the impact of various parameters within a single step. This was beyond the scope of this study; we attempted to optimize the steps that were used according to extant literature in order to investigate the impact of each step in the pipeline. Different parameters would assuredly change the impact of each step, but comprehensively investigating all permutations of parameters within a pipeline would overwhelm the statistical models. Further, certain combinations of parameters would be unjustifiable from the literature and inappropriate to use. It would be relevant in a subsequent study to investigate various arguments in order to see if the impact of each step in a pipeline could be optimized. Third, only three bilateral ROIs were included in the analysis. A small number of ROIs were specifically used in order to avoid overwhelming the statistical models. The hippocampus was selected as it is known to be sensitive to pre-processing in registration-based pipelines due to a similar intensity signature with the amygdala and the thin alveus which delineates anterior hippocampus from posterior/ventral amygdala [35, 61]. The putamen was selected as we considered it to have good white-matter boundaries with other subcortical gray-matter structures. The middle temporal gyrus was selected both in order to have a cortical structure as well as to assess the impact of skull-stripping in this pipeline as it has been established [27, 60, 62] that skull-stripping impacts cortical volume. Fourth, a single scanning sequence was used in investigating the effect of pre-processing step on ROI volumes. Using the established OASIS dataset was done in order to decrease potential sources of noise in the data. It is likely that differing scanning sequences would produce different amounts of noise, which may produce an interaction with the pre-processing pipeline. This, however, was also beyond the scope of this study, and warrants further investigation. Fifth, the pipelines in this study used only registration-based segmentation. This was done because it was the intention of this preliminary study to look at the impact on the data of various steps within a single pipeline. Different pipelines (e.g. non-registration-based) would undoubtedly have different variances associated with them and would be worth investigating.

### 4.3 Recommendations

As each step in the pre-processing pipeline produced differences in either volume or variance, we recommend calculating the percent variance error (PVE) correction, to account for the differential effects that these pre-processing steps have on the data, before comparing analyses. Volumetric and morphometric studies reporting significance with a small difference in voxel numbers are readily available [63, 64–74], and may be false positives as the data consists, in part, of unaccounted noise, some of which stems from pre-processing. A difference of a few tens of voxels in sensitive longitudinal or multi-group studies may well be accounted for by the upper and lower bounds provided by the PVE correction, particularly if there are differences in pre-processing steps. Additional noise is probable if there are further differences in scanning sequences, MRI scanners, software suites, pipeline steps, and arguments used.

We also recommend, echoing Tustison et al., [75], that a detailed description of the pre-processing used be provided in order to clarify any impact that pre-processing may have had on the overall outcome of the analysis. Additionally, the inclusion of pre-processing steps will help make replication an easier task [76, 77].
